# Rapid and Sensitive Digital Droplet PCR Assays for Detecting HPV16 DNA in Liquid Biopsies

**DOI:** 10.1002/jmv.70146

**Published:** 2024-12-29

**Authors:** Suet Kee Loo, Jian Feng, Carly Reeder, Danny Azmi Elias, Zhongping Xu, Yufei Huang, Kevin J. Contrera, Jose P. Zevallos, Robert Ferris, Shou‐Jiang Gao

**Affiliations:** ^1^ Cancer Virology Program, UPMC Hillman Cancer Center University of Pittsburgh School of Medicine Pittsburgh Pennsylvania USA; ^2^ Department of Microbiology and Molecular Genetics University of Pittsburgh School of Medicine Pittsburgh Pennsylvania USA; ^3^ UPMC Hillman Cancer Center University of Pittsburgh Pittsburgh Pennsylvania USA; ^4^ Department of Otolaryngology University of Pittsburgh Pittsburgh Pennsylvania USA; ^5^ Department of Medicine University of Pittsburgh School of Medicine Pittsburgh Pennsylvania USA

**Keywords:** ddPCR, digital PCR assay, direct detection, HPV16 detection, liquid biopsies, rapid and sensitive assay

## Abstract

The combination of cell‐free DNA (cfDNA) and digital droplet PCR (ddPCR) has significantly advanced the noninvasive screening, diagnosis, and monitoring of diseases, enabling highly sensitive and absolute quantification of target nucleic acids even in the presence of high background DNA. However, widespread adoption of ddPCR is hindered by higher costs, extended processing times, and the requirement for cfDNA purification, which adds expense and variability. To address these limitations, we developed two optimized ddPCR‐based assays tailored for enhanced sensitivity, cost‐efficiency, and ease of use. Our highly sensitive ddPCR assay for human papilloma virus (HPV)16 DNA detection in purified cfDNA from liquid biopsies from head and neck cancer patients significantly improved sensitivity by increasing cfDNA concentration by 8.5‐fold, sample volume loading by 22‐fold, and total cfDNA amount tested by 1200‐fold without the need for restriction enzyme digestion. In parallel, we established a rapid ddPCR assay using unpurified cfDNA processed by heat treatment and centrifugation, achieving detection concordance rates of 55.6%, 66.7%, and 95.8% for plasma, serum, and surgical drain fluid (SDF), respectively, compared to purified cfDNA. Together, these complementary workflows, one optimized for unpurified cfDNA and the other for purified cfDNA, make ddPCR detection of specific targets in cfDNA more cost‐effective, time‐efficient, and standardizable across laboratories, paving the way for broader adoption in clinical diagnostics.

## 1 Introduction

Significant progress has been made in cancer biology and oncology in recent years; however, effective detection, diagnosis, and monitoring of cancer remain challenging [1]. Conventional methods often involve exposure to harmful radiation or invasive procedures to obtain samples through biopsy. In some cases, pre‐cancerous lesions or small tumors remain undetected by radiographic imaging, allowing disease progression before patients experience symptoms or treatment is initiated, sometimes missing the optimal therapeutic window [2, 3, 4]. Additionally, biopsies may be unsuccessful when tumors are located in hard‐to‐reach areas, often requiring multiple attempts to obtain sufficient tissue for diagnosis, which can be both risky and time‐consuming [4]. Even during remission, many patients undergo periodic radiographic imaging, which poses long‐term health risks and may not be ideal for continual monitoring [5, 6].

Cell‐free DNA (cfDNA) refers to DNA fragments present in the noncellular fraction of body fluids, including peripheral blood (e.g., serum and plasma), cerebrospinal fluid, saliva, pleural fluid, urine, and postoperative serosanguineous fluid, or surgical drain fluid (SDF) [7, 8]. cfDNA can originate from various cellular sources, including normal cells, tumor cells, fetal tissue, and infectious agents such as viruses [9]. cfDNA can be released through various processes such as apoptosis and necrosis from normal and diseased or tumor cells [10, 11, 12, 13]. cfDNA exists as freely circulating DNA, DNA within extracellular vesicles, or DNA encapsulated in viral particles [9, 14, 15]. The presence of cfDNA in body fluids allows for minimally invasive sampling, diagnosis, and disease monitoring through liquid biopsy, which is easier, less harmful, and more convenient than conventional tissue biopsies and radiographic imaging techniques [7, 9]. Since its discovery in 1948 by Mandel and Metais [16], liquid biopsy has demonstrated applications across various stages of disease, including screening, diagnosis, and monitoring [9]. Indeed, previous studies have shown that cfDNA levels in healthy individuals are generally low under normal conditions, but increase significantly in response to abnormal conditions such as tissue stress, surgery, injury, inflammation, tumor presence, and even exercise [7, 17, 18]. Patients with elevated cfDNA levels, such as those with colorectal cancer, tend to have shorter survival rates compared to those with lower cfDNA levels [19], while those with HPV‐associated oropharyngeal squamous cell carcinoma have higher disease recurrence [20]. Tumor‐derived cfDNA can vary widely within the total cfDNA in body fluids, constituting anywhere from less than 0.1% to over 90% [18, 21].

Digital droplet PCR (ddPCR) is an advanced type of digital PCR assay that partitions the reaction mixture into thousands of water‐in‐oil droplets, significantly enhancing sensitivity compared to conventional digital PCR [22, 23]. Initially developed by Hindson et al. ddPCR enables absolute quantitation of nucleic acids without requiring a standard curve [22]. Since its introduction, ddPCR has been widely adopted in medical applications, particularly for detecting rare targets amidst high background levels of nontarget sequences or wild‐type genes [24, 25]. The first FDA‐approved ddPCR assay, granted in 2019, monitors chronic myeloid leukemia by quantifying BCR‐ABL1 transcript levels, an indicator of disease progression and treatment response, in total RNA from whole blood [26].

ddPCR can detect mutant alleles at frequencies below 0.001% in a wild‐type background, offering a sensitivity that is over 1000 times greater than conventional PCR methods [22, 27]. This ultra‐sensitive detection capability has broad applications, including identifying mutations in prenatal testing, such as fetal‐specific alleles in maternal blood [28, 29], and detecting oncogenic mutations like *KRAS* and *EGFR* in lung cancer [30]. ddPCR is also instrumental in identifying pathogens responsible for diseases, including severe acute respiratory syndrome coronavirus 2 (SARS‐CoV‐2) [31], tuberculosis [32], and Epstein‐Barr virus (EBV) in lymphomas [33]. More recently, ddPCR assays have been developed for HPV52, 58, 56, 6, 11, 16, 18, 33, and 45 [34], and used in cervical and vulvar cancers as well as HPV‐associated oropharyngeal squamous cell carcinoma (HPV+ OPSCC) [35, 36].

Current methods for detecting targets in liquid biopsies, such as plasma and serum, require cfDNA extraction and restriction digestion before ddPCR analysis [27, 37, 38]. This approach is costly, time‐intensive, and laborious, often resulting in lower cfDNA yields, which decreases the likelihood of target detection [12]. Variability in cfDNA yield depending on the extraction method further limits data consistency and comparability across laboratories [39, 40, 41].

In this study, we aimed to develop fast and sensitive ddPCR assays to detect HPV16 in various liquid biopsy samples from patients with head and neck cancer. HPV16 accounts for about 90% of HPV‐positive head and neck cancers [42, 43], which have a better prognosis than HPV‐negative cases [44, 45]. Thus, accurate HPV status diagnosis is essential for informing therapeutic decisions. Our assays could facilitate this process, offering a practical tool for clinical applications. Notably, we optimized the cfDNA isolation method to increase cfDNA concentration, and thus improving the chance of detecting the target in a single ddPCR reaction. By reducing the volume of eluent for cfDNA by fivefold, we improved the droplet count by nearly 8.5‐fold in purified cfDNA. In addition, we demonstrated that cfDNA can be eluted with PCR reaction solution, thus maximizing and increasing sample volume loading by 22‐fold. We also demonstrated robust HPV16 detection even with 1200 ng of background cfDNA without the need for restrictive digestion. Furthermore, we developed a direct detection assay of HPV16 DNA in liquid biopsies without the need for cfDNA extraction. Direct ddPCR on unpurified cfDNA yielded results comparable to purified cfDNA, with detection concordance rates of nearly 55.6% in plasma, 66.7% in serum, and 95.8% in SDF. Overall, we have developed rapid and sensitive detection assays that are more cost‐ and time‐efficient, suitable for use with different types of liquid biopsies from patients. These streamlined, cost‐ and time‐efficient ddPCR assays are adaptable across liquid biopsy types and particularly useful for cases with low target fractions or viral loads, providing a valuable tool for accurate HPV16 detection in clinical settings.

## 2 Methods and Materials

### 2.1 Sample Collection

This study utilized liquid biopsies, including plasma, serum, and SDF collected from head and neck cancer patients treated at the University of Pittsburgh Medical Center (UPMC). Specimen collection followed an approved University of Pittsburgh research protocol (IRB# 99‐069) in compliance with the Declaration of Helsinki. Plasma samples were obtained from 46 patients, serum from 20 patients, and SDF from 26 patients, including 20 paired plasma‐serum and 20 paired plasma‐SDF cases. Plasma was isolated from whole blood collected in tubes with anticoagulants (EDTA or heparin), while serum was isolated from whole blood in silicone‐coated tubes with clot activator. SDF was directly collected in tubes. All collected liquid biopsies were stored at −80°C until analysis. Patients included those with tumors positive for HPV DNA or P16, following College of American Pathologists guidelines [46], and HPV DNA‐negative cases as controls.

### 2.2 Cell‐Free DNA Extraction

cfDNA was extracted from C3.43 cell supernatant, plasma, serum, and SDF using the QIAamp Circulating Nucleic Acid Kit (Qiagen, Cat. No. 55114) following the manufacturer's instructions. Unless specified otherwise, cfDNA was eluted with elution buffer at a volume ratio of 1:50 relative to the volume of the liquid biopsy.

### 2.3 DNA Extraction From Cell Line

Whole‐cell DNA was extracted from the Kaposi's sarcoma‐associated herpesvirus (KSHV) infected primary rat embryonic metanephric mesenchymal precursor cells [47] using the QIAamp DNA Mini Kit (51304, Qiagen) according to the manufacturer's instructions.

### 2.4 Droplet Digital PCR (ddPCR) Detection of HPV16

Detection of HPV16 in cfDNA extracted from liquid biopsy samples or unpurified cfDNA was conducted using the QX200 Droplet Digital PCR System (Bio‐Rad) and ddPCR Supermix for Probes (Bio‐Rad, Cat. No. 1863024) according to the manufacturer's instructions. The primers and probes used were as follows: forward primer 5′‐TGTTTCAGGACCCACAGGAG‐3′, reverse primer 5′‐TGTTGCTTGCAGTACACACA‐3′, and probe FAM‐5’‐ ACCACAGTTATGCACAGAGCTGCAAAC‐3′‐HEX. The primers targeted the E6 region of HPV16 DNA, amplifying a product of 106 bp. The optimal annealing temperature was determined to be 56.5°C, based on the fluorescence signal difference between positive and negative controls and the number of plasmids detected (Figure S1).

Quantification of positive droplets was performed using the ddPCR direct quantification method, with a manually set fluorescence amplitude cut‐off of 0 across all tests using QX Manager software (Bio‐Rad, version 1.1). A sample was defined as positive for HPV16 if at least one positive droplet was detected in the assay.

### 2.5 Sample Preparation for ddPCR Detection of HPV16 DNA in Unpurified cfDNA

Before ddPCR reaction, unless otherwise stated, all plasma and serum were subjected to 5x dilution with ddPCR Supermix for Probes (1863024, Bio‐Rad) followed by 3 min of heat‐treatment in 100°C and rapid cooling on ice for 30 s before centrifugation at 12 000 rpm at 4°C for 10 min. Notably, due to the loss of samples to evaporation and aggregation during heat treatment, we started with higher volumes of sample. For triplicate ddPCR reaction, we diluted 5 μL of plasma or serum with 20 μL of ddPCR Supermix and subjected the diluted sample to heat‐treatment. In the final ddPCR reaction mix, 5 μL of diluted and processed plasma or serum, which contained 1 μL of initial plasma or serum, were used per 25 μL ddPCR reaction.

Unless otherwise stated, SDF samples were subjected to 3x dilution with water and underwent the same procedures as plasma and serum thereafter. For triplicate ddPCR reaction, 5 μL of SDF was diluted with 10 μL of water before heat‐treatment. In a single reaction, 3 μL of diluted and processed SDF consisted of 1 μL of initial SDF was used in the final 25 μL ddPCR reaction mix.

Control samples without heat treatment were subjected to the same procedures as described above, excluding the 3 min heat treatment step.

### 2.6 Plasmid DNA Spike‐In

The pBABE‐Puro plasmid containing HPV16 E6 and E7 genes (HPV16 plasmid DNA) [48], kindly provided by Dr. Xuefeng Liu, was used as a spike‐in control for the ddPCR reactions. This plasmid was directly added to HPV16‐negative plasma, serum, or SDF samples to mimic HPV16‐positive specimens. The plasmid copy number was calculated using a publicly available copy number calculator, and serial dilutions were performed to achieve the desired concentrations for use as spike‐in controls.

### 2.7 Statistical Analysis

For unpaired samples, statistical significance was assessed using the Mann–Whitney test or two‐tailed unpaired *t*‐test. For paired samples, the Wilcoxon matched‐pairs signed rank test was applied. For categorical comparisons of paired samples, statistical significance was determined using Fisher's exact test.

## 3 Results

### 3.1 Sensitive Detection of HPV16 DNA in the Presence of High Background Undigested DNA

**Figure 1:**
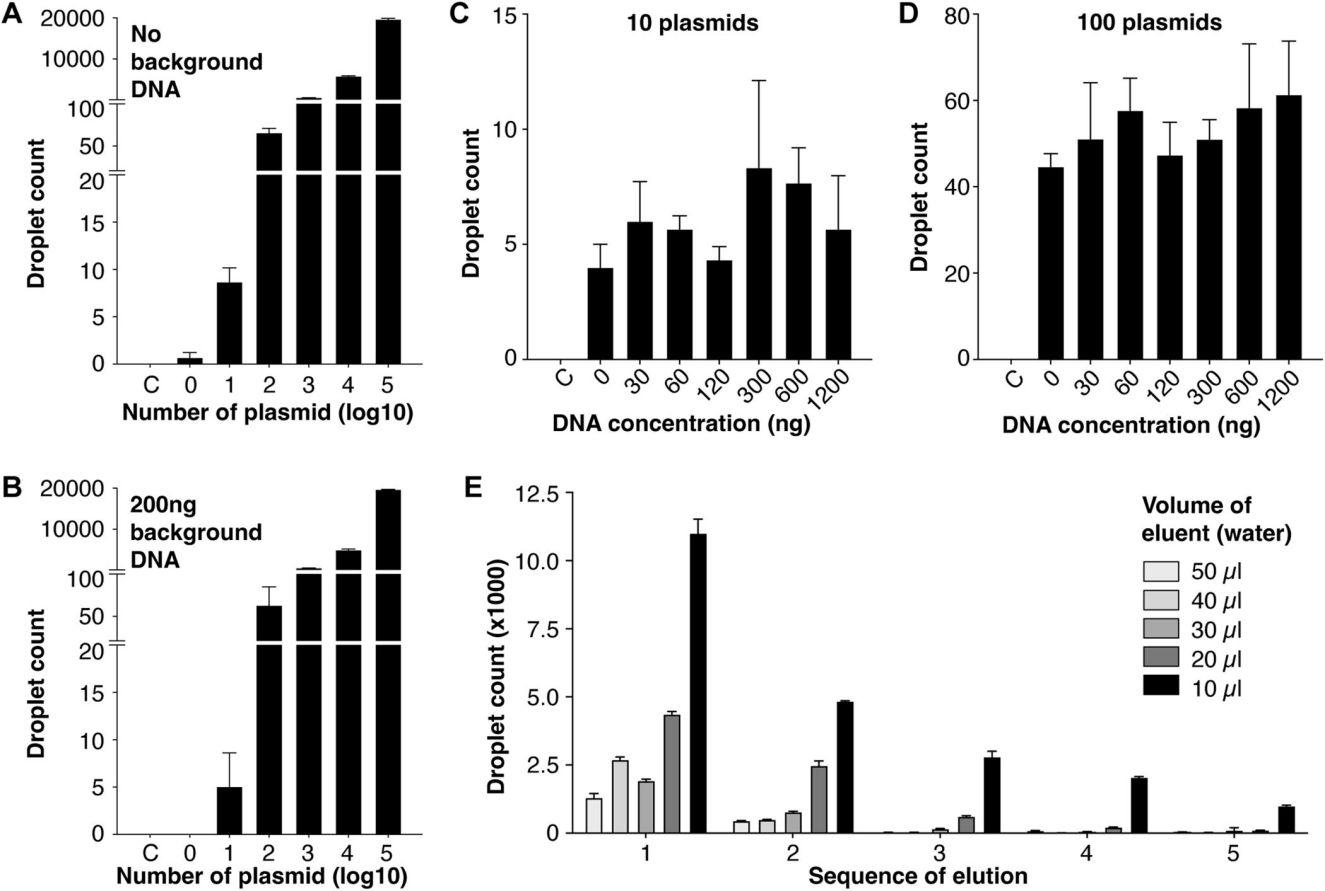
Optimization of HPV16 DNA detection in purified cfDNA. (A, B) HPV16 DNA droplet count following HPV16 plasmid DNA spike‐in ranging from 0 to 100 000 copies in the absence of background DNA (A) or presence of 200 ng DNA in the background (B). (C, D) HPV16 DNA droplet count following 10 (C) or 100 (D) copies of HPV16 plasmid DNA spiked‐in in ddPCR reaction mix containing background DNA ranging from 30 to 1200 ng. Reaction mix without HPV16 plasmid DNA and background DNA was used as negative control. (E) HPV16 DNA droplet count in cfDNA extracted from supernatant of C3.43 cells in culture. cfDNA was eluted with different volumes of water as eluent, ranging from 10 to 50 μL, for up to five times. C: Negative control.

We aimed to investigate the feasibility of detecting HPV16 DNA in total cfDNA extracted from liquid biopsies without the use of restriction digestion. We optimized a ddPCR assay for HPV16 DNA with an annealing temperature of 56.5°C (Figure S1). As reported previously, cfDNA is typically highly fragmented, with an average fragment size of around 180 bp, and its concentration varies between patients, ranging from 10 to 1200 ng/mL in plasma, with a mean concentration of approximately 219 ng/mL [10]. To evaluate HPV16 DNA detection across different cfDNA concentrations, we spiked HPV16 DNA into varying amounts of whole cell DNA from 0 to 1200 ng. By omitting restriction digestion, we minimized additional cfDNA fragmentation, thereby maximizing the potential for HPV16 DNA detection. To establish detection sensitivity, we created a gradient control of HPV16 DNA copy numbers through serial spiking. Without background cfDNA, the assay had 67% probability of detecting a single copy of HPV16 DNA (Figure 1A). In the presence of 200 ng of background cfDNA, the detection limit was an average of five copies of HPV16 DNA for reaction spiked‐in with 10 copies of HPV16 DNA, compared to the detection limit of an average of 8.7 copies without background DNA (Figure 1A,B). For HPV16 DNA quantities exceeding 100 copies, detection limits were unaffected by the presence or absence of background cfDNA (Figure 1A,B). Notably, the detection of 10 and 100 copies of HPV16 DNA was consistent in the presence of varying background cfDNA amounts, up to 1200 ng (Figure 1C,D). These results indicate that using up to 1200 ng of cfDNA per reaction, instead of the standard 1 ng in a regular PCR reaction, can significantly enhance detection sensitivity for clinical samples that usually have a wide range of concentrations.

Since up to 1200 ng of background DNA did not affect detection sensitivity, we aimed to enhance the concentration of cfDNA purified from samples by reducing the elution buffer volume, thereby increasing the number of positive droplets detected. This modification has the potential to significantly improve detection sensitivity in samples with very low cfDNA concentrations, such as urine, by effectively concentrating cfDNA from samples for analysis.

We extracted cfDNA from the supernatant of cultured C3.43 cells and tested eluent volumes ranging from 10 to 50 µL, with 50 µL being the volume recommended by the manufacturer for the cfDNA extraction kit. Each extraction column was eluted with the same volume up to five times. Results showed that 10 µL of eluent yielded the highest cfDNA concentration across all five elutions (Figure S2A). The first elution typically retrieved 43%–50% of the total cfDNA recovered from all five elutions, with subsequent elutions continuing to yield cfDNA, although concentrations decreased by 50%–65% with each additional elution.

Compared to the manufacturer‐recommended 50 µL, a 10 µL elution volume increased cfDNA concentration by nearly 3.5‐fold across the five elutions (Figure S2A). Similarly, using 10 µL yielded the highest positive droplet count in each elution (Figure 1E). In the first elution, 10 µL of eluent resulted in an average of around 11 000 positive droplets per ddPCR reaction, compared to approximately 1300 positive droplets with 50 µL, an increase of nearly 8.5‐fold. Generally, compared to 10 µL of eluent, elution with volumes of 20 µL or more reduced positive droplet counts by at least half in all five elutions (Figure 1E).

In a 25 µL ddPCR reaction mix, the ddPCR Supermix constitutes 12.5 µL, allowing for a maximum specimen volume of approximately 9.6 µL. To further increase the specimen volume in a single reaction, we eluted cfDNA with ddPCR Supermix instead of water, raising the potential total specimen volume to 22.1 µL per reaction. We then compared assay performance between using water and ddPCR Supermix as the cfDNA eluent. For the first three elutions, no significant difference was observed in the number of positive droplets detected between the two eluents (Figure S2B). Surprisingly, almost twofold more droplets were detected with Supermix than water in the subsequent elutions. Thus, up to 22.1 µL of cfDNA sample per reaction can be loaded, compared to the standard 1 µL in a regular PCR reaction, potentially enhancing detection sensitivity for clinical samples by an additional 22‐fold. These results indicate that ddPCR Supermix can serve as an eluent when a higher volume of cfDNA is required in a single ddPCR reaction, thereby enhancing the likelihood of detecting the target of interest.

In summary, by increasing the concentration of purified cfDNA, maximizing the sample volume loaded, and enhancing the total cfDNA amount tested in a single reaction, we achieved detection sensitivity improvements of 8.5‐, 22‐, and 1200‐fold, respectively, resulting in an overall sensitivity increase of 224 400‐fold.

### 3.2 Detection of HPV16 DNA in cfDNA Extracted From Different Types of Human Liquid Biopsies

Using the optimized conditions, we extracted cfDNA from various HPV DNA‐positive liquid biopsy types, including plasma, serum, and SDF (purified cfDNA). Matched specimens were available for 20 patients (Set 1) with serum and plasma obtained from the same individuals, and for another 20 patients (Set 2) with matched plasma and SDF. To standardize elution conditions across all liquid biopsies, cfDNA was eluted using an eluent‐to‐sample ratio of 1:50, with a minimum eluent volume of 10 µL. For HPV16 DNA detection, 200–300 ng of cfDNA was used whenever sufficient cfDNA quantity was available.

**Figure 2:**
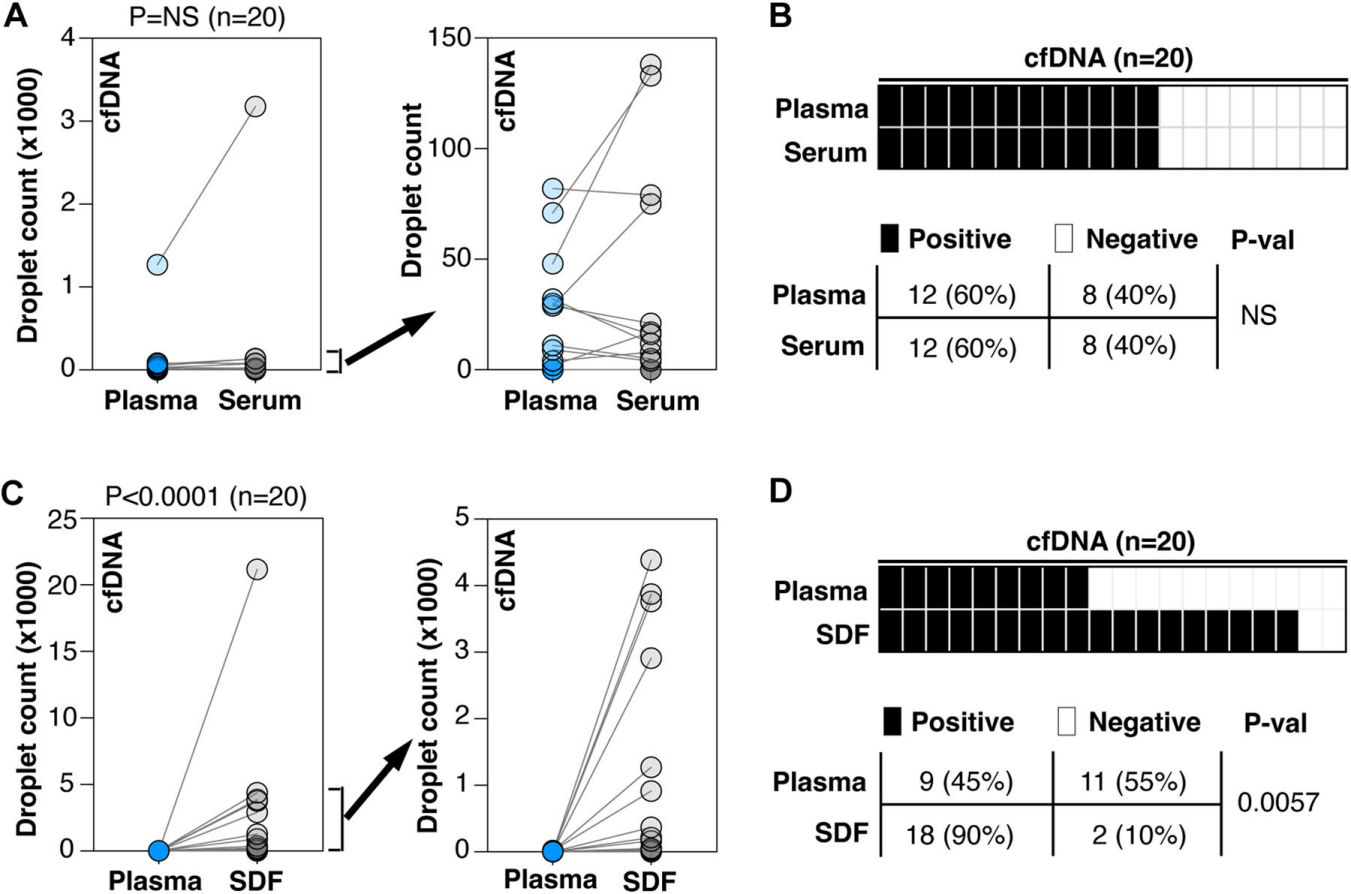
Detection of HPV16 DNA in purified cfDNA. (A, B) Purified cfDNA from paired plasma and serum samples showing the numbers of detected droplets (A) and detection of HPV16‐positive cases (B). Right panel selectively showed the droplet counts for paired cases with droplet counts less than 150 in the left panel (A). (C, D) Purified cfDNA from paired plasma and surgical drain fluid (SDF) samples showing the numbers of detected droplets (C) and detection of HPV16‐positive cases (D). Right panel selectively showed the droplet counts for paired cases with droplet counts less than 5000 droplets in the left panel (C). A sample is considered positive with the presence of a minimum of one HPV16 DNA‐positive droplet in a single ddPCR reaction (B, D) with black box represents HPV16 DNA‐positive sample and white box represents HPV16 DNA‐negative sample. *p*‐values were assessed by Wilcoxon matched‐pairs signed rank test with *p* < 0.05 considered having a significant difference (A, C). *p*‐values were assessed by Fisher's exact test with *p* < 0.05 considered having a significant difference (B, D). *p*‐val: *p*‐value; NS, not significant.

In the first set of specimens, the number of HPV16 DNA‐positive droplets detected in purified plasma cfDNA and purified serum cfDNA was comparable (Figure 2A). Patients with detectable HPV16 DNA in purified plasma cfDNA also had HPV16 DNA present in purified serum cfDNA. Similarly, patients without detectable HPV16 DNA in purified plasma cfDNA also showed absence of HPV16 DNA in their serum (Figure 2B). In the second set of specimens, purified SDF cfDNA showed a significantly higher positive droplet count than purified plasma cfDNA (*p* < 0.0001, Figure 2C). Additionally, there was a significantly higher number of patients with detectable HPV16 DNA in purified SDF cfDNA compared to purified plasma cfDNA (*p* = 0.0057, Figure 2D). Similar to controls, the baseline fluorescence of cfDNA extracted from plasma, serum, or SDF, showed base fluorescence below 0 (Figure S3A,B).

### 3.3 Direct Detection of HPV16 DNA in Unpurified Liquid Biopsy

**Figure 3:**
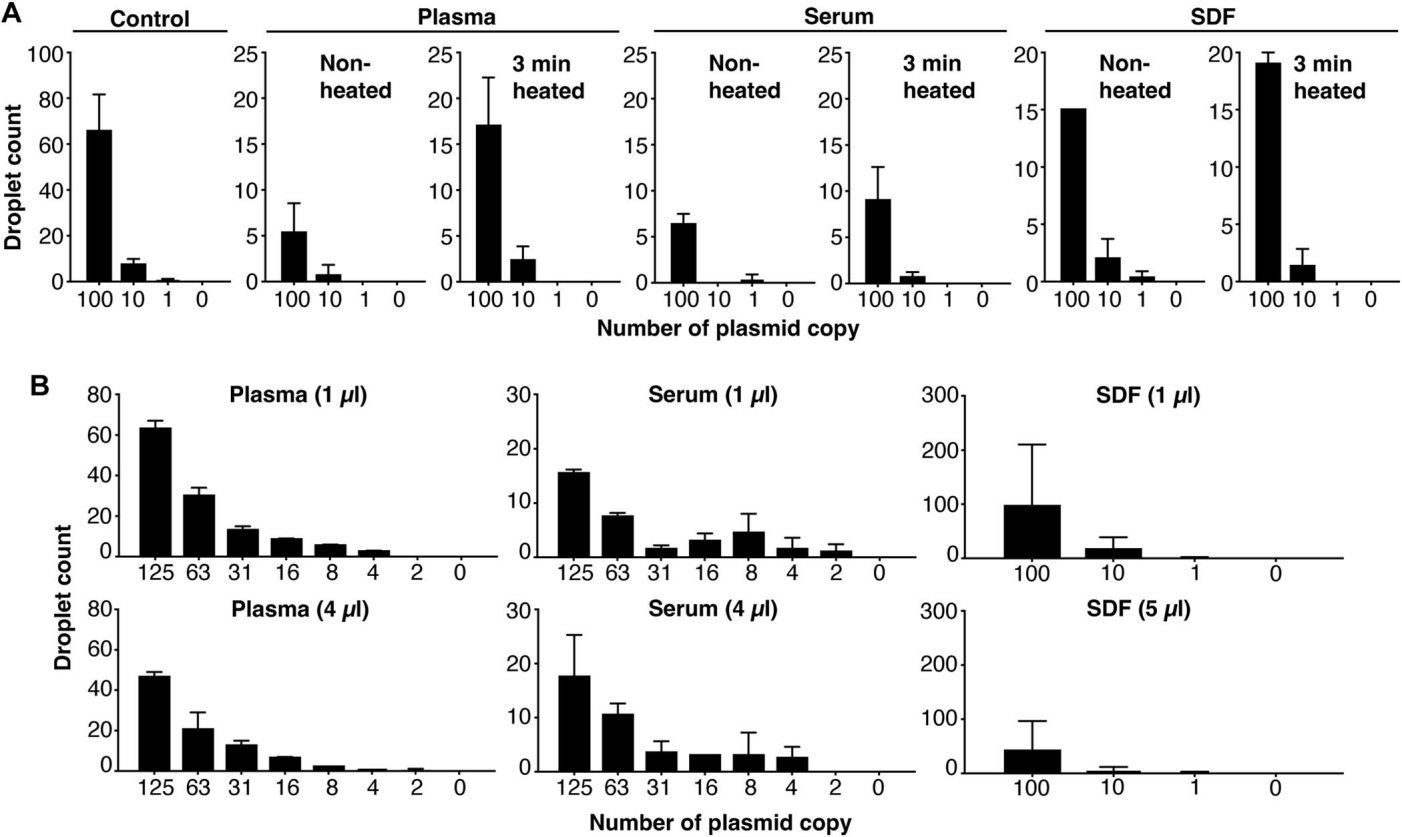
Optimization of ddPCR detection of HPV16 DNA in unpurified cfDNA samples. (A) Detection of HPV16 DNA droplets in raw non‐heated liquid biopsies and liquid biopsies that underwent 3 min of heat treatment at 100°C for plasma, serum and surgical drain fluid (SDF), respectively. Control comprises of ddPCR reactions without any liquid biopsies. (B) Detection of HPV16 DNA droplets in 1 and 4 μL (plasma and serum) or 5 μL (SDF) of diluted and processed liquid biopsies from plasma, serum and SDF samples, respectively.

Given that cfDNA purification is a relatively time‐consuming and costly process, we evaluated the feasibility of directly detecting HPV16 DNA in plasma, serum, and SDF without cfDNA extraction (unpurified cfDNA). To assess potential sample inhibition, we spiked HPV16 plasmid DNA into HPV16 DNA‐negative liquid biopsies and compared HPV16 DNA‐positive droplet counts between non‐heated unpurified specimens and unpurified specimens subjected to 3 min of heat treatment. In samples spiked with 100 HPV16 plasmids, heated plasma, serum, and SDF samples showed average droplet counts of 17, 9, and 19, respectively, and were substantially higher than their nonheated counterparts, which displayed average droplet counts of 5, 6, and 15, respectively (Figure 3A). Heat treatment did not affect the baseline fluorescence of plasma, serum, or SDF, as all tested specimens, including non‐heated samples and controls, showed base fluorescence below 0 (Figures S4A–C).

We further examined the effect of different volumes of heated specimens in the HPV16 DNA detection assay. We tested 1 µL of sample in a ddPCR reaction. Specifically, we diluted 5 μL of plasma, serum or SDF sample with 20 μL of ddPCR Supermix and subjected the diluted sample to heat treatment. In the final ddPCR reaction mix, 5 μL of diluted and processed plasma or serum, which contained 1 μL of initial plasma or serum, were used per 25 μL ddPCR reaction. We also tested 4 µL of plasma or serum sample in a ddPCR reaction by diluting 20 µL of sample with 5 µL of ddPCR Supermix and subjected the diluted sample to heat‐treatment. In the final ddPCR reaction, 5 µL of diluted and processed plasma or serum which contained 4 µL of plasma or serum, were used in the final ddPCR reaction. For SDF sample, we tested 5 µL of sample in the final ddPCR reaction. We diluted 25 µL of SDF with 10 µL of water and tested 7 µL of diluted and processed sample in the final ddPCR reaction (Figure 3B).

Our results demonstrated that using 1 µL of heated plasma or SDF yielded a higher droplet count compared to using 4 or 5 µL of heated plasma or SDF, respectively (Figure 3B). However, 1 and 4 µL of heated serum returned with similar droplet counts. Hence, we used 1 µL of heated unpurified cfDNA in subsequent experiments.

### 3.4 Difference in HPV16 DNA Level Between Unpurified and Purified Liquid Biopsies

Using the optimized conditions for HPV16 DNA detection in unpurified cfDNA, we next analyzed unpurified and purified cfDNA from the same samples. As expected, the number of positive droplets was significantly higher in purified cfDNA from plasma (*p* = 0.0003), serum (*p* = 0.0185) and SDF (*p* = 0.0439) than their unpurified counterparts due to the concentrating effect of the purification process (Figure S5). Additionally, cfDNA extracted from plasma and serum showed a 20% higher detection rate of cases than unpurified plasma and serum cfDNA. In contrast, approximately similar numbers of positive cases were detected for the SDF samples using unpurified and purified cfDNA (88% vs. 93% in Figure S5).

**Figure 4:**
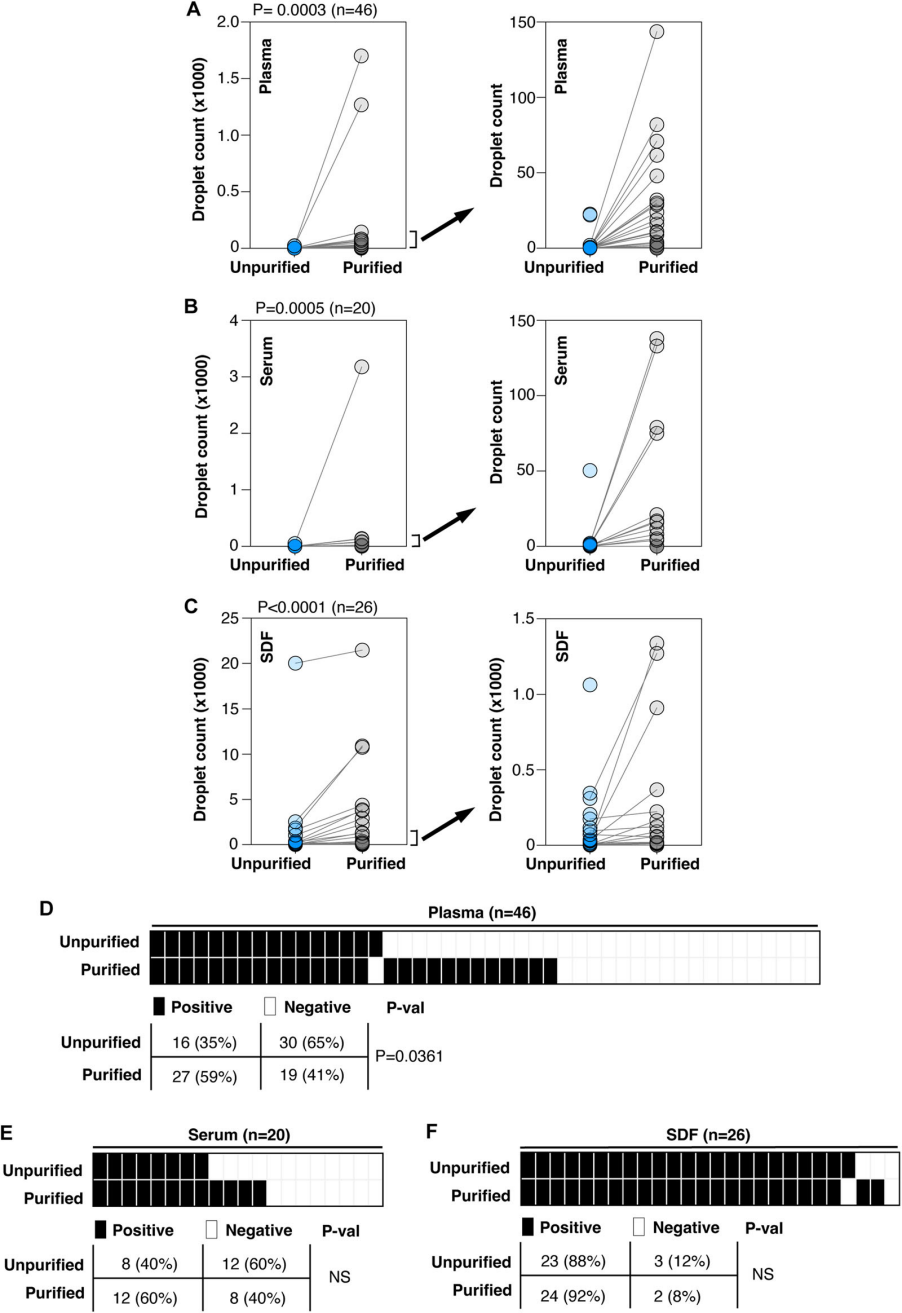
Comparisons of detection of HPV16 DNA in unpurified and purified cfDNA samples. (A–C) Detection of HPV16 DNA droplets in paired unpurified samples and purified cfDNA samples from (A) plasma (*n* = 46), (B) serum (*n* = 20), and (C) surgical drain fluid (SDF, *n* = 26). *p*‐values were assessed via Wilcoxon matched‐pairs signed rank test. *p* < 0.05 is considered as significantly different. The right panels selectively showed the droplet counts for paired cases with droplet counts of less than 150 droplets (A, B) or 1500 droplets (C) in the left panels. (D–F) Detection of HPV16‐positive cases in plasma (C), serum (E), or SDF (F) samples. A case is considered positive with the presence of a minimum of one HPV16 DNA droplet in a single ddPCR reaction. Each box represents one specimen. Stacked boxes represent both unpurified cfDNA and purified cfDNA from the same liquid biopsy. Black box represents HPV16 DNA positive specimen and white box represents HPV16 DNA negative specimen. *p*‐values were assessed via Fisher's exact test with *p* < 0.05 considered having a significant difference. *p*‐val: *p*‐value; NS, not significant.

A similar trend was observed when comparing droplet counts between unpurified and purified cfDNA from plasma, serum, or SDF collected from the same patients (Figure 4). Purified cfDNA consistently demonstrated significantly higher droplet counts in plasma (*p* = 0.0003, Figure 4A), serum (*p* = 0.0005, Figure 4B), and SDF (*p* < 0.0001, Figure 4C) compared to their unpurified counterparts. Of those positive for HPV16 using purified cfDNA, 55.6%, 66.7%, and 95.8% were also positive using unpurified cDNA in plasma, serum, and SDF, respectively (Figure 4D–F). Only plasma samples showed a statistical difference when purified and unpurified cfDNA were compared (Figure 4D). Clearly, minimal difference in HPV16 detection rate was found between purified and unpurified SDF samples (Figure 4F). Interestingly, one case of HPV16‐positive unpurified plasma cfDNA sample was negative when examined with its purified plasma cfDNA (Figure 4D), and one case of HPV16‐positive unpurified SDF cfDNA sample was negative when examined with its purified plasma cfDNA (Figure 4F).

## 4 Discussion

The discovery of cfDNA has significantly transformed the medical field, enabling disease detection, diagnosis, and monitoring to be conducted in a minimally invasive manner [49]. Our study demonstrated that the existing protocol for cfDNA extraction and ddPCR detection can be optimized to improve both the efficiency and sensitivity of the assay (Figure 1A–E). We assessed the assay's sensitivity across various amounts of background DNA (0–1200 ng), mimicking clinical samples with similar cfDNA quantities [10].

In our workflow, we diverged from the kit manufacturer's protocol by omitting the use of restriction enzymes. Our rationale was that restriction enzymes could further fragment the already fragmented cfDNA and viral DNA, potentially decreasing the likelihood of positive detection, particularly in cases where the target mutant cfDNA or viral DNA is present in very low fractions amidst a high background of wildtype or host cfDNA. Supporting this approach, a study by Weaver et al. found that ddPCR detection accuracy remained 100% concordant with or without restriction enzymes [50], which is in agreement with our findings. In our study, the number of detected plasmids remained consistent both in the absence and presence of up to 1200 ng of undigested background DNA (Figure 1A–D). These results indicate that as high as 1200 ng DNA could be used in a single ddPCR reaction without the need of restriction digestion, thus maximizing the amount of input cfDNA in a single reaction.

We demonstrated that reducing the elution volume during cfDNA extraction by five‐fold increased the detection limit by up to 8.5‐fold (Figure 1E), with a minimum tested elution volume of 10 µL. Additionally, we found that cfDNA could be eluted with ddPCR Supermix without compromising detection sensitivity (Figure S2B). Using ddPCR Supermix as an eluent increases the maximum sample volume that a 25 µL ddPCR reaction can accommodate to 22.1 µL. Compared to the 1 µL sample loading in a standard PCR reaction, this adjustment allows for a 22‐fold increase in the amount of cfDNA tested, thereby enhancing sensitivity in a single reaction.

In this study, we used a liquid biopsy to eluent ratio of 50:1; however, a higher ratio could be applied to further increase detection sensitivity, especially for samples with low cfDNA levels, such as urine. This higher concentration of extracted cfDNA enhances the likelihood of detecting targets that are present in very low amounts. We applied our optimized workflow to actual patient samples and observed comparable performance for purified cfDNA from plasma and serum samples while purified cfDNA from SDF samples had the highest detection rates (Figure 2A–D). This finding is expected, as SDF is collected directly from the acute wound bed and regional lymph nodes post‐surgery, unlike plasma or serum from blood, which circulates and may be more distant from the tumor site, potentially reducing the viral load and chance of positive detection [8].

Despite these improvements, we found cases that were tested negative for HPV16 in purified cfDNA from all three types of samples. We reasoned that these cases could either be positive for other HPV variants other than HPV16 or contain HPV16 DNA below the detection threshold of the assay despite the improved detection sensitivity.

In short, by increasing cfDNA concentration by 8.5‐fold, sample volume loading by 22‐fold, and total cfDNA amount tested by 1200‐fold, all without the need for restriction enzyme digestion, we achieved up to a 224 400‐fold increase in detection sensitivity utilizing purified cfDNA from liquid biopsies. Thus, we have streamlined the cfDNA extraction process to create a workflow that is more sensitive and standardized, reducing variability and enabling consistent outcomes across laboratories.

Next, we developed a rapid detection assay using unpurified cfDNA. While cfDNA isolation is typically performed before amplification and detection by ddPCR, this process is costly, time‐consuming, and yields variable results depending on the method and reagents used [40, 51]. Studies report cfDNA yields in plasma varying from 1.6 to 28.1 ng/mL and in serum from 4.5 to 184 ng/mL [40, 51], suggesting that different isolation methods can impact detection accuracy and potentially increase false negatives, especially with low cfDNA yields. Our results demonstrate that positive detection is achievable with unpurified cfDNA.

Unpurified plasma, however, contains PCR inhibitors such as immunoglobulin G, lactoferrin, and heparin [52, 53, 54]. We hypothesized that ddPCR detection with unpurified samples could be feasible by reducing or removing these inhibitors. By subjecting diluted specimens to heat treatment and centrifugation, we observed aggregates (likely composed of proteins and other inhibitors), which are known to be heat‐labile. Using the supernatant for ddPCR, we found that heated samples yielded higher droplet counts than unheated ones, indicating reduced inhibitor levels (Figure 3A). Nonetheless, droplet counts remained lower in spiked plasma, serum, or SDF than the buffer controls, suggesting that heat treatment does not completely eliminate inhibitors (Figure 3A). Increasing the sample volume also reduced positive droplet counts, likely due to the increased concentrations of inhibitors (Figure 3B).

cfDNA extraction remains widely used to reduce inhibitors and concentrate cfDNA before ddPCR. Consistently, purified cfDNA showed higher positive droplet counts across all liquid biopsies (Figure 4A–C), as each ddPCR reaction used a concentrated cfDNA sample, amplifying detection sensitivity by approximately 50‐fold compared to unpurified cfDNA in this current study. However, despite its lower cfDNA concentration, unpurified cfDNA displayed good concordance with purified cfDNA, with concordance rates of 55.6%, 66.7%, and 95.8% for plasma, serum, and SDF, respectively (Figure 4D–F). Thus, this fast assay could be used to identify a substantial fraction of positive cases, particularly in SDF specimens, while negative cases could subsequently undergo DNA purification for more sensitive detection.

In summary, we have optimized the ddPCR detection protocol for HPV16 in liquid biopsies by developing a rapid assay and a highly sensitive assay. Future improvements could include integrating a multiplex assay to detect multiple HPV variants and expanding testing to a wider range of sample types, such as urine, saliva, and other body fluids, to assess the assays' versatility across diverse liquid biopsy samples. By eliminating steps like cfDNA isolation and restriction digestion, and improving cfDNA purification, our ddPCR‐based HPV16 detection assays are now more time‐, cost‐, and labor‐efficient and easier to standardize across laboratories, ultimately enabling more consistent and reliable results.

## Author Contributions

Shou‐Jiang Gao and Robert Ferris conceived the project. Shou‐Jiang Gao designed, supervised, and managed the project. Suet Kee Loo and Jian Feng performed the experiments. Carly Reeder, Danny Azmi Elias, and Zhongping Xu identified the subjects and samples, and assembled them for the experiments. Kevin J. Contrera, Jose P. Zevallos, and Robert Ferris conducted the clinical study. Yufei Huang, Kevin J. Contrera, Jose P. Zevallos, Robert Ferris, and Shou‐Jiang Gao interpreted the data and participated in the discussions throughout the analysis. Suet Kee Loo and Shou‐Jiang Gao wrote the manuscript with input from all the authors. All the authors read, reviewed, and approved the manuscript.

## Conflicts of Interest

The authors declare no conflicts of interests.

## Supporting information


**Supplementary information** The online version contains supplementary material available at:

**Supplementary Figure 1:** Representative ddPCR droplet plot showing fluorescence amplitude of droplets (Top panel) and bar graph showing the corresponding droplet counts for different annealing temperature of PCR amplification tested (Lower panel). Blue and grew droplets in the droplet plot represents HPV16 DNA‐positive and ‐negative droplets, respectively (Top panel) with purple line representing fluorescence amplitude of 0 and Y‐axis representing fluorescence amplitude.

**Supplementary Figure 2: (A)** Concentrations of cfDNA extracted from supernatants of C3.43 cells in culture eluted with different volumes of water as eluent, ranging from 10 μl to 50 μl for 5 times. **(B)** HPV16 DNA droplet count in cfDNA samples extracted from supernatants of C3.43 cells in culture eluted with 20 μl water and 20 μl ddPCR Supermix for 5 times. P‐values were assessed via two‐tailed unpaired t‐test with P< 0.05 considered having a significant difference. NS: Not significant.

**Supplementary Figure 3:** ddPCR droplet plots showing fluorescence amplitude of HPV16 DNA‐positive or ‐negative droplets in purified cfDNA samples. (**A**) Detection of HPV16 DNA in cfDNA purified from plasma or serum samples ran in the same ddPCR 96‐well plate, and droplets were read at the same time. Controls are ddPCR reaction mix with HPV16 DNA spiked‐in, and the same control was used for both purified plasma and serum. For plasma, P1 to P8 represent 8 representative plasma samples from 8 patients from which the cfDNAs were purified from. For serum, S1 to S8 represent 8 representative serum samples from the same 8 patients as plasma, from which the cfDNAs were purified from. **(B)** Detection of HPV16 DNA in cfDNA purified from surgical drain fluid (SDF) samples. Controls are ddPCR reaction mix with HPV16 DNA spiked‐in. D1 to D8 represent 8 representative SDF samples from 8 patients from which the cfDNAs were purified from. Red lines represent fluorescent amplitude of 0. Blue droplets represent HPV16 DNA‐positive droplets while grey droplets represent HPV16 DNA‐negative droplets.

**Supplementary Figure 4:** ddPCR droplet plots showing fluorescent amplitude of HPV16 DNA‐positive or ‐negative droplets in non‐heated and heated unpurified samples. (**A**) Plasma, **(B)** Serum, and **(C)** Surgical drain fluid (SDF). Controls are ddPCR reaction mix without unpurified liquid biopsies. All the control, unpurified plasma and serum samples were run in the same ddPCR 96‐well plate and droplets were read at the same time. Thus, the same control was used for both unpurified plasma and serum samples (**A** and **B**). Red lines represent fluorescent amplitude of 0. Blue droplets represent HPV16 DNA‐positive droplets while grey droplets represent HPV16 DNA‐negative droplets.

**Supplementary Figure 5: Detection of HPV16 DNA droplet counts in unpurified and purified cfDNA samples.** Differences in droplet counts of unpaired unpurified and purified cfDNA were shown for serum, plasma and surgical drain fluid (SDF) samples, respectively. Percentages represent cases that are positive for HPV16 DNA based on the presence of a minimum of one HPV16 DNA droplet. P‐values were assessed via Mann Whitney Test with P< 0.05 considered having a significant difference.

## Data Availability

The data supporting findings are available upon request.
