# Supplementary figures and images for: Rapid and Sensitive Digital Droplet PCR Assays for Detecting HPV16 DNA in Liquid Biopsies
Source: J Med Virol. 2024 Dec 29;97(1):e70146. doi: 10.1002/jmv.70146 (PMC11683180; doi:10.1002/jmv.70146)

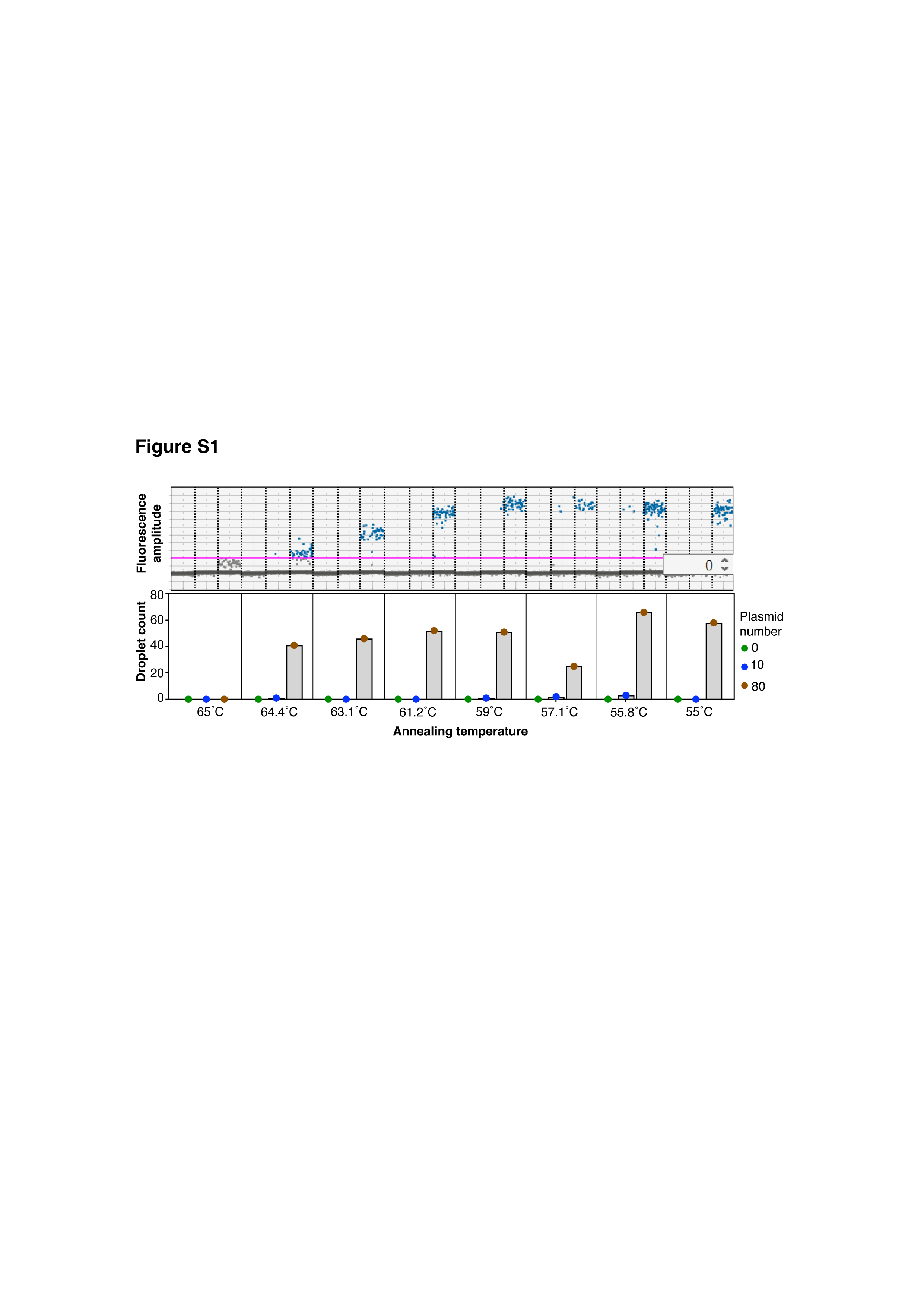

Supplement: Supplementary file 1 — Supplementary Figure 1: Representative ddPCR droplet plot showing fluorescence amplitude of droplets (Top panel) and bar graph showing the corresponding droplet counts for different annealing temperature of PCR amplification tested (Lower panel). Blue and grew droplets in the droplet plot represents HPV16 DNA‐positive and ‐negative droplets, respectively (Top panel) with purple line representing fluorescence amplitude of 0 and Y‐axis representing fluorescence amplitude. [file JMV-97-e70146-s001.tif]

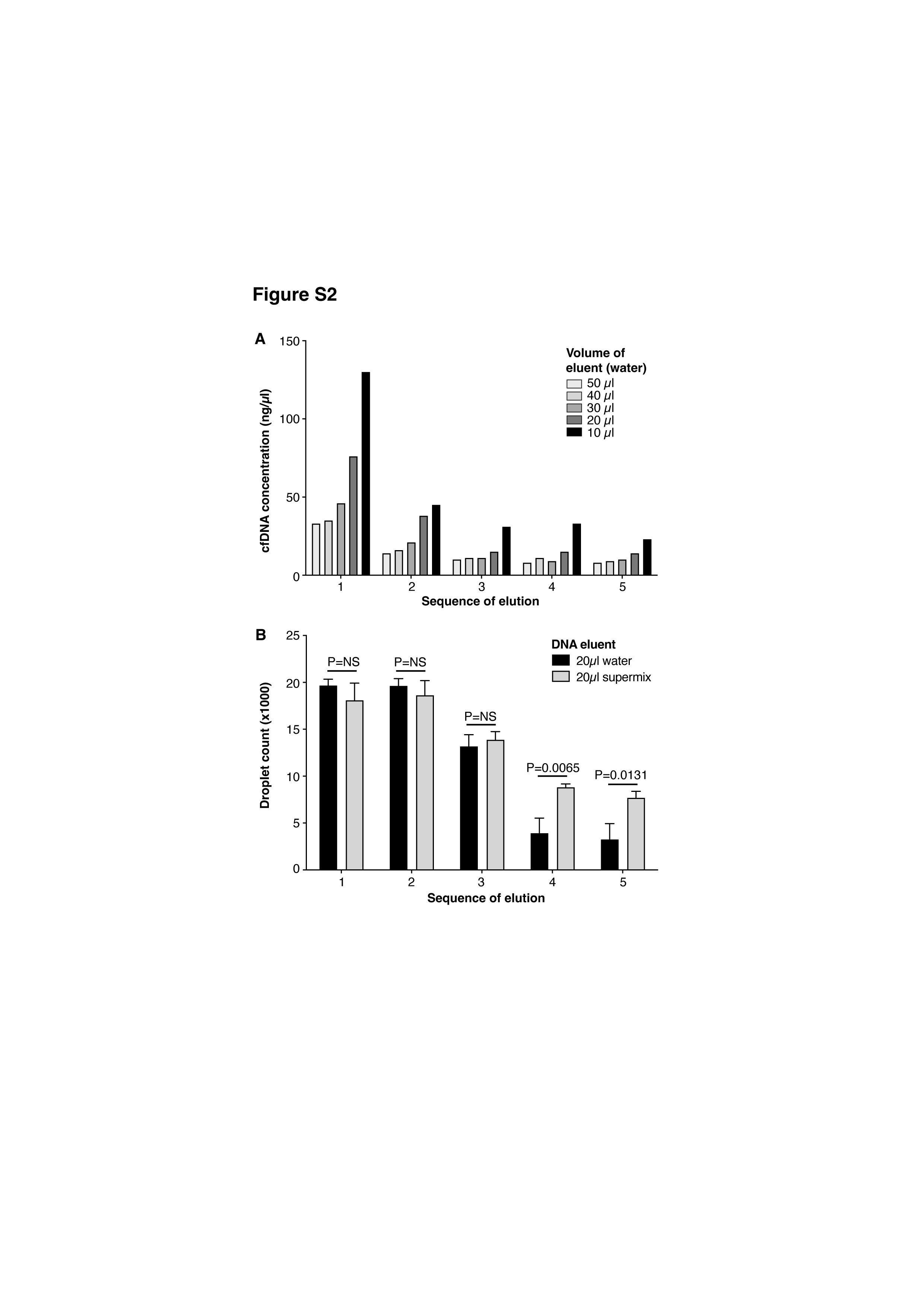

Supplement: Supplementary file 2 — Supplementary Figure 2: (A) Concentrations of cfDNA extracted from supernatants of C3.43 cells in culture eluted with different volumes of water as eluent, ranging from 10 μl to 50 μl for 5 times. (B) HPV16 DNA droplet count in cfDNA samples extracted from supernatants of C3.43 cells in culture eluted with 20 μl water and 20 μl ddPCR Supermix for 5 times. P‐values were assessed via two‐tailed unpaired t‐test with P< 0.05 considered having a significant difference. NS: Not significant. [file JMV-97-e70146-s004.tif]

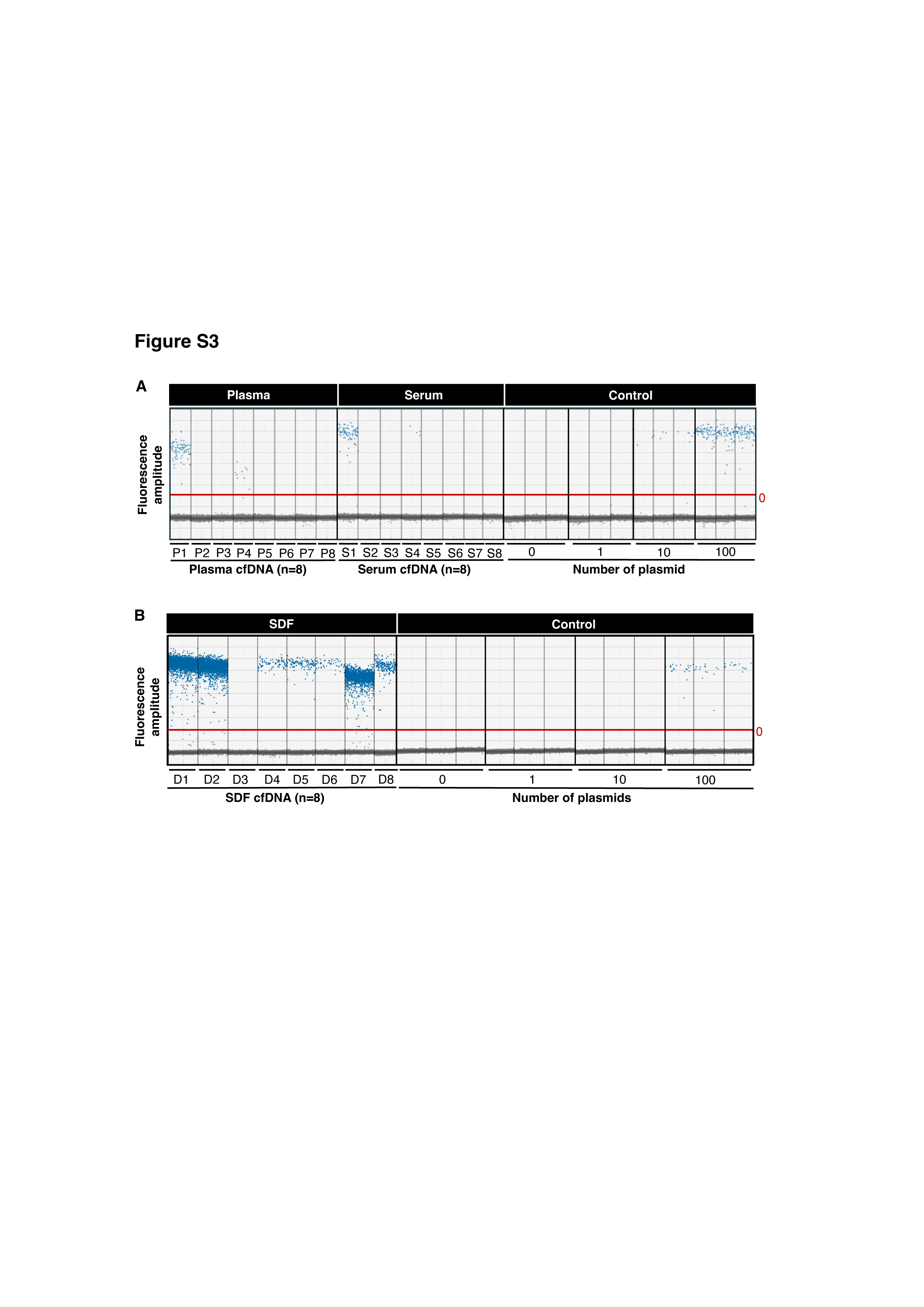

Supplement: Supplementary file 3 — Supplementary Figure 3: ddPCR droplet plots showing fluorescence amplitude of HPV16 DNA‐positive or ‐negative droplets in purified cfDNA samples. (A) Detection of HPV16 DNA in cfDNA purified from plasma or serum samples ran in the same ddPCR 96‐well plate, and droplets were read at the same time. Controls are ddPCR reaction mix with HPV16 DNA spiked‐in, and the same control was used for both purified plasma and serum. For plasma, P1 to P8 represent 8 representative plasma samples from 8 patients from which the cfDNAs were purified from. For serum, S1 to S8 represent 8 representative serum samples from the same 8 patients as plasma, from which the cfDNAs were purified from. (B) Detection of HPV16 DNA in cfDNA purified from surgical drain fluid (SDF) samples. Controls are ddPCR reaction mix with HPV16 DNA spiked‐in. D1 to D8 represent 8 representative SDF samples from 8 patients from which the cfDNAs were purified from. Red lines represent fluorescent amplitude of 0. Blue droplets represent HPV16 DNA‐positive droplets while grey droplets represent HPV16 DNA‐negative droplets. [file JMV-97-e70146-s005.tif]

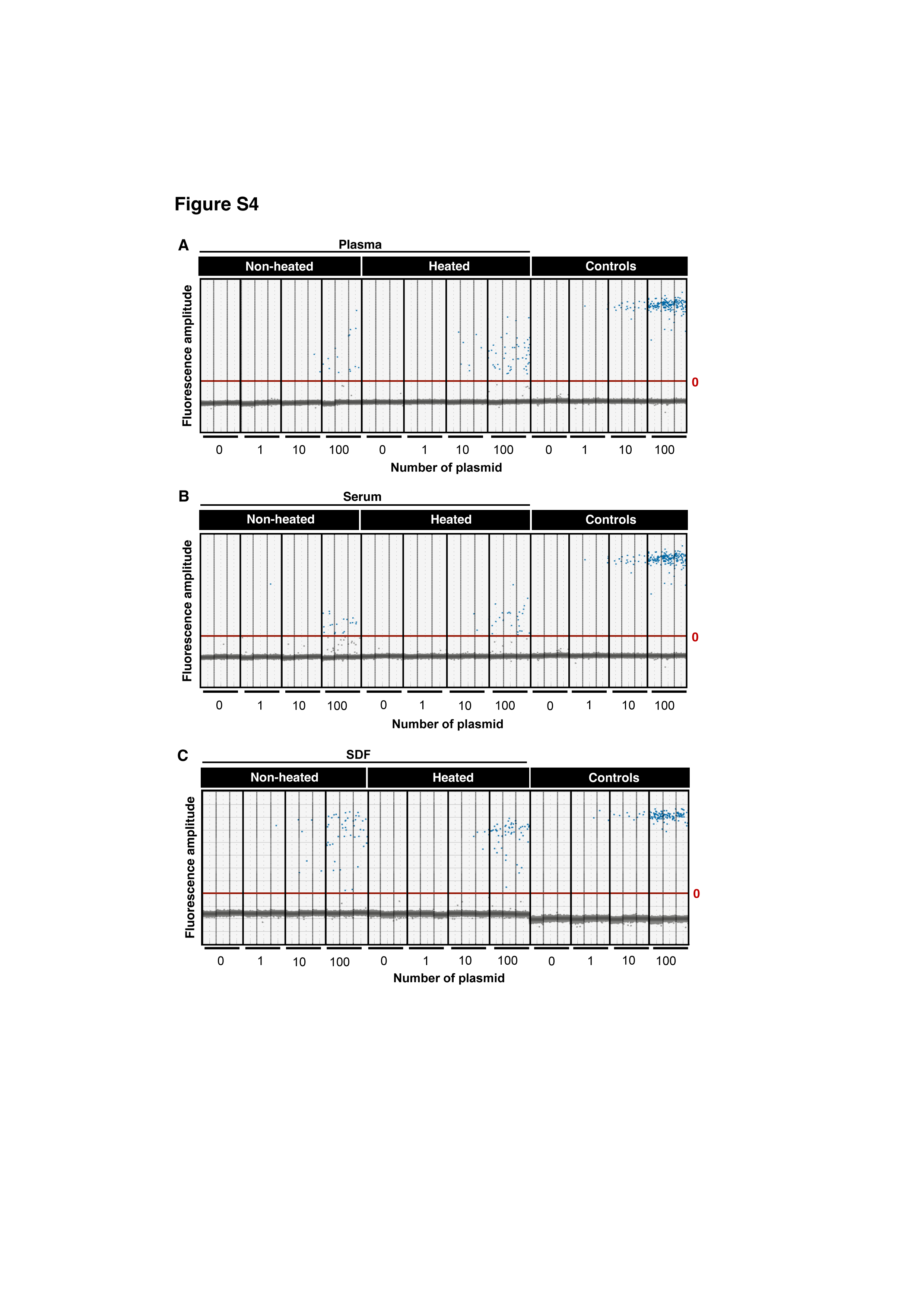

Supplement: Supplementary file 4 — Supplementary Figure 4: ddPCR droplet plots showing fluorescent amplitude of HPV16 DNA‐positive or ‐negative droplets in non‐heated and heated unpurified samples. (A) Plasma, (B) Serum, and (C) Surgical drain fluid (SDF). Controls are ddPCR reaction mix without unpurified liquid biopsies. All the control, unpurified plasma and serum samples were run in the same ddPCR 96‐well plate and droplets were read at the same time. Thus, the same control was used for both unpurified plasma and serum samples (A and B). Red lines represent fluorescent amplitude of 0. Blue droplets represent HPV16 DNA‐positive droplets while grey droplets represent HPV16 DNA‐negative droplets. [file JMV-97-e70146-s003.tif]

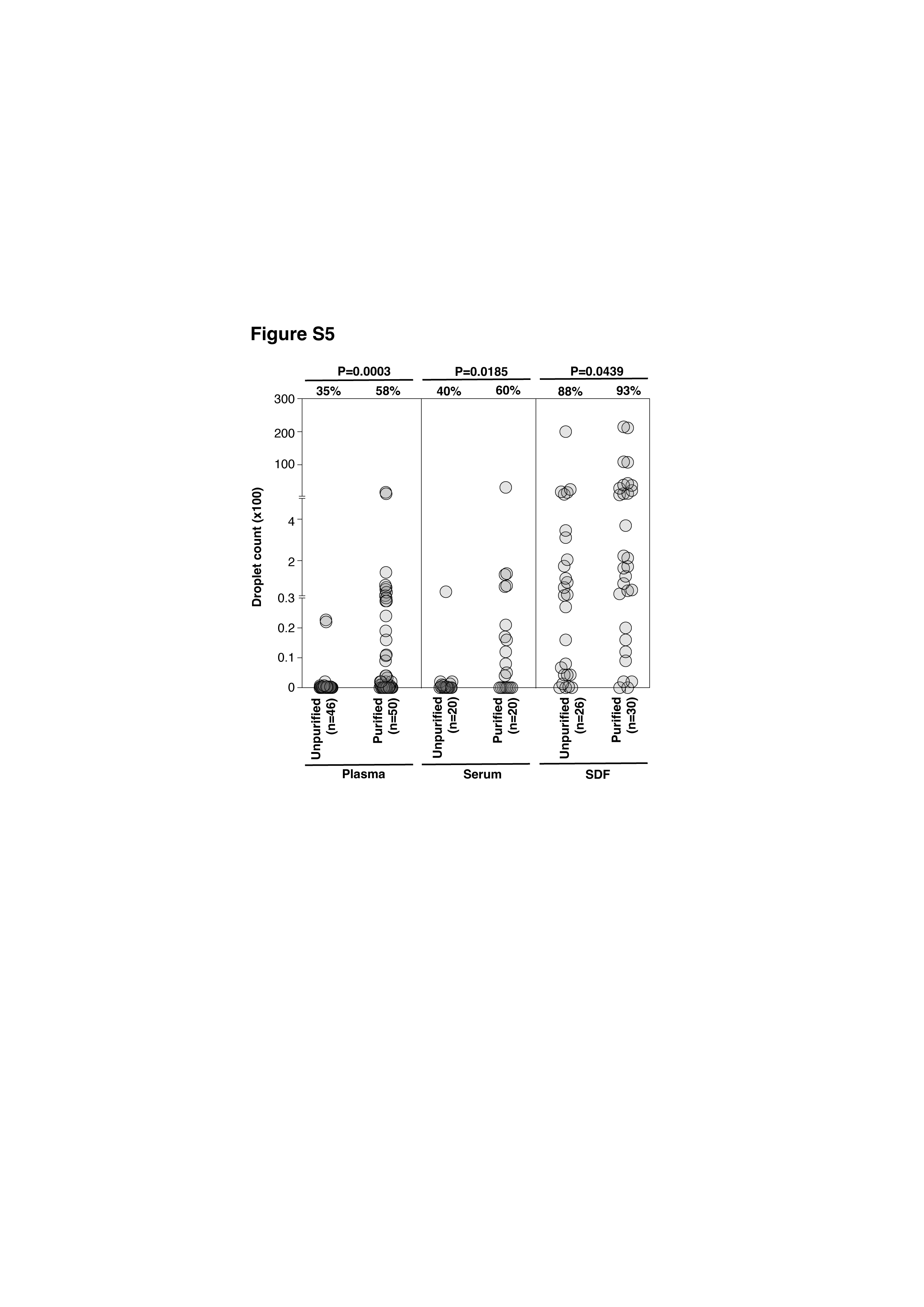

Supplement: Supplementary file 5 — Supplementary Figure 5: Detection of HPV16 DNA droplet counts in unpurified and purified cfDNA samples. Differences in droplet counts of unpaired unpurified and purified cfDNA were shown for serum, plasma and surgical drain fluid (SDF) samples, respectively. Percentages represent cases that are positive for HPV16 DNA based on the presence of a minimum of one HPV16 DNA droplet. P‐values were assessed via Mann Whitney Test with P< 0.05 considered having a significant difference. [file JMV-97-e70146-s002.tif]
